# Distal colonic atresia: a case report

**DOI:** 10.1093/jscr/rjad335

**Published:** 2023-06-12

**Authors:** Tom Allert, Vera Schellerer

**Affiliations:** Department of Pediatric Surgery, University Medicine Greifswald, Greifswald, Germany; Department of Pediatric Surgery, University Medicine Greifswald, Greifswald, Germany

## Abstract

Colonic atresia (CA) is a rare disease with an incidence range between one of 20 000 and one of 66 000 live births. Most CA are located within the proximal colon; distal CA are even rarer. Because of its rarity, another case shall be described herewith. A 37th week of pregnancy born child was noticed occurring multiple vomiting, a distended abdomen and additional whitish-bloody stool shortly thereafter. In the first operation, a double-barrel stoma was created. After sufficient weight gain and alignment of the stoma ends, a secondary anastomosis was created in the child after 2 months. The diagnosis can be made reliably on the basis of an X-ray and leads to a good outcome with prompt surgical intervention. However, accompanying malformations should always be considered.

## INTRODUCTION

Colonic atresia (CA) is a rare disease [1] of the newborn defined as a complete interruption of the colon [2]. The incidence ranges between one of 20 000 [3] and one of 66 000 live births [4]. Most CA are located within the proximal colon; distal CA are even rarer. Clinically, patients are conspicuous by persistent vomiting, a distended abdomen and absent meconium excretion [5]. The only therapy is surgical intervention [4, 6]. Because of the rarity of this pathology, another case will be described herewith.

## CASE REPORT

The child was born in the 37th week of pregnancy with a birth weight of 2270 g. The pregnancy was uncomplicated. Because of postpartal persisting vomiting, a distended abdomen and additional whitish-bloody stool, an abdominal X-ray was performed. This revealed distended small and large bowel loops with evidence of an obstruction of passage in the distal colon. Emergency operation was performed on the second day of life. Intraoperatively, a complete atresia of the descending colon with massively dilated transverse and ascending colon and hypoplastic sigmoid colon and rectum was found. Atresia was resected and a double colostomy was created because a primary anastomosis was not possible because of a caliber difference of the partial ends of 5:1. Trial excisions from the oral stoma and the aboral blind stoma demonstrated physiological nerve innervation. A gradual mechanical dilation of the aboral stoma was performed postoperatively. Because of the slowly closing oral stoma recurrent dilatation was performed. According to a simultaneous *Clostridium difficile* colonization of the intestine, oral therapy with metronidazole and flushing of the aboral stoma with vancomycin was performed 1 week before the stomas were re-displaced. Subsequently, after a total of 2 months with a weight auf 3580 g, the stomas were repositioned with an end-to-end anastomosis. The gradual diet buildup was well tolerated.

## DISCUSSION

The cause of CA continues to be debated in research. Direct triggers of this disease, such as intestinal stricture, internal hernia, or intestinal obstruction, are rarely found to cause CA [7]. It is thereby discussed that these trigger a vascular event and thus promote atresia [8]. Recent research data provided evidence for genetic involvement [9]. Furthermore, there is a connection to many other malformations such as omphalocele, gastroschisis, exomphalos, vesicointestinal fistula, imperforate anus and choledochal cyst [10]. The association between Hirschsprung’s disease and CA is very rare, but can be found in the literature [11]. A common classification of intestinal atresia is the modified classification according to Grosfeld *et al*. [12]. In this classification, Type I shows a mucosal atresia. In Type II, the atretic ends of the intestine are joined by a band of fibrous tissue. In Type IIIa the atretic ends are separated by a V-shaped mesenteric defect. Inserted is the Type IIIb lesion consisting of an apple skin deformity with proximal CA and a single retrograde blood supply to the distal end of the bowel. A Type IV is present as multiple atresias. Type IIIa is the most common type of atresia [13]. The distribution pattern of atresia across the colon is not homogeneous. In a larger review, Davenport *et al*. [4] describe 118 infants with occurrence of atresia at 28% of the ascending colon, 3% of the hepatic flexure, 23% of the transverse colon, 25% of the splenic flexure and 20% of the descending and sigmoid colon.

There are several options for surgical therapy. Whereas earlier studies advocated primary anastomosing of the atresia proximal to the splenic flexure and secondary anastomosing distal to it, the location of the CA plays a more subordinate role in more recent studies. Various authors have also achieved very good results with primary anastomosing [4, 5]. However, primary anastomosing may lead to higher complication rates. On the one hand, this is because of unrecognized pathologies of the distal atresia end [14], but also because of the massive caliber difference of both ends. In the case described this was 5:1 between the oral and aboral end, so that primary anastomosing was not a therapeutic option.

Outcome in CA now appears to be very good. Recent reports show a mortality rate of 0% [5]. However, the risk increases with further associated malformations [15].

## CONCLUSION

CA can have a very good outcome if they are recognized promptly on the basis of their symptoms, such as absent meconium excretion and persistent vomiting. The choice of therapy is anastomosis, with primary and secondary anastomosis being equally important options. Nevertheless, in the case of a present CA, concomitant anomalies should be searched for, which may influence the overall outcome.

## Data Availability

No new data were generated or analysed in support of this research.
